# Impact of Hypothyroidism in Pregnancy on Feto-Maternal Outcomes: A Prospective Observational Study

**DOI:** 10.7759/cureus.74494

**Published:** 2024-11-26

**Authors:** Rutuja Khawale, Sujata R Kanetkar, Mahendra Patil

**Affiliations:** 1 Department of Pathology, Krishna Institute of Medical Sciences, Krishna Vishwa Vidyapeeth (Deemed To Be University), Karad, IND

**Keywords:** complications, fetal outcome, hypothyroidism, maternal outcome, pregnancy

## Abstract

Introduction

Hypothyroidism represents an endocrine disorder marked by the insufficient production of hormones by the thyroid gland, with significant effects on bodily functions. Its occurrence during pregnancy is of particular concern due to its profound effects on both maternal and fetal health outcomes.

Aim

To study the impact of hypothyroidism in pregnancy and its correlation with feto-maternal outcomes.

Methodology

The present study is a two-year prospective observational study carried out at a tertiary care hospital from July 2022 to June 2024. A total of 350 antenatal women with singleton pregnancies and without any pre-existing medical disorder were screened. Serum thyroid stimulating hormone (TSH) was evaluated with a cut-off value of 4.0 mIU/L. If serum TSH was abnormal, free thyroxine (FT4) and free triiodothyronine (FT3) levels were assessed. The participants were categorized into three groups designated as euthyroid, overt hypothyroidism, and subclinical hypothyroidism. TSH was periodically re-evaluated at 16 weeks, 20 weeks, and 32 weeks. All patients were monitored up to the point of delivery, allowing for comparison of outcomes across the three groups.

Results

The prevalence of hypothyroidism in the present study was 11.14% (n=39). Eight percent (n=28) of the cases had subclinical hypothyroidism whereas 3.14% (n=11) of cases had overt hypothyroidism. Hypothyroidism was more common in the 26-30 years age group. There was a higher incidence of hypothyroidism in multigravida patients. Lower segment caesarean section (LSCS) was the most common mode of delivery in the women with hypothyroidism. Maternal outcomes included preeclampsia, eclampsia, anemia, gestational hypertension, gestational diabetes mellitus (GDM), spontaneous miscarriage, preterm labor, oligohydramnios, etc. The fetal outcomes included intrauterine growth restriction (IUGR), low birth weight (LBW) and fetal distress.

Conclusion

Early diagnosis and adequate treatment of maternal hypothyroidism leads to successful pregnancy outcomes. Therefore, universal thyroid screening in pregnancy is recommended in order to prevent maternal and fetal complications.

## Introduction

Hypothyroidism is an endocrine disorder marked by the insufficient production of thyroid hormones by the thyroid gland, with significant effects on bodily functions. Its occurrence during pregnancy is of particular concern due to its profound effects on both maternal and fetal health outcomes [1].

The incidence of hypothyroidism in India is notably higher in women and increases with age. Overt hypothyroidism affects 3-4.5% of the general population, while subclinical hypothyroidism, which presents with elevated thyroid stimulating hormone (TSH) but normal thyroid hormone levels, impacts about 6.45-9% of individuals. The risk factors for hypothyroidism encompass gender, age, autoimmune conditions, a history of thyroid dysfunction, and iodine scarcity [2,3].

The symptoms of hypothyroidism are often vague like fatigue, weight gain, cold intolerance, constipation, dry skin, hair loss, and menstrual irregularities. When left untreated in pregnant women, it may lead to adverse maternal and fetal outcomes. The diagnosis hinges on serum TSH and free thyroxine (FT4) levels. However, pregnancy introduces complexities due to physiological adjustments affecting hormone levels. Hence, pregnancy-specific reference ranges are necessary for accurate interpretation [4,5].

The physiological changes during pregnancy complicate the detection and screening of hypothyroidism as many symptoms like weight gain, menstrual irregularities, fatigue, hair loss, etc. are common to both conditions. Pregnancy may also affect thyroid hormone levels, leading many experts to recommend universal screening to facilitate early detection and intervention [4,5]. This approach aims to avoid the risk of complications associated with the dysfunction of the thyroid gland in pregnancy.

The impact of hypothyroidism on pregnancy is profound. It is associated with various adverse outcomes such as elevated risk of miscarriage, gestational hypertension, and placental abnormalities in the mother as well as low birth weight (LBW) and neurodevelopmental impairments in the child [1]. These considerations highlight the critical importance of adequate screening and management of hypothyroidism in pregnant women to minimize these risks. This comprehensive approach establishes the crucial importance of studying the impact of hypothyroidism impact on feto-maternal outcomes, demonstrating the vital role of universal screening in advancing maternal and neonatal health.

## Materials and methods

Study design

The study was conducted over two years (July 2022 to June 2024) at Krishna Hospital and Medical Research Centre, Karad, Maharashtra, a tertiary care hospital equipped with the essential facilities and equipment needed to conduct the study efficiently. The study was designed as a prospective observational study and the protocol was approved by the Institutional Ethics Committee of Krishna Institute of Medical Sciences, Krishna Vishwa Vidyapeeth (Deemed To Be University), Karad (approval no. 282/2021-2022).

Sample size

The sample size was determined to be 350 cases by the formula n=4pq/L^2^ where, 'p' is prevalence, q=100-p, and 'L' is the permissible error. For the present study, p=5.6%, q=94.4%, and L=3%.

Inclusion and exclusion criteria 

Antenatal women with singleton pregnancies and without any pre-existing medical disorders were included in the study. Women diagnosed with hypothyroidism before pregnancy, women on drugs that alter thyroid function (e.g., amiodarone, tricyclic antidepressants, selective serotonin reuptake inhibitors, and metformin), multi-fetal gestation, known cases of chronic diseases like diabetes, hypertension, renal, or cardiovascular diseases and women diagnosed with hyperthyroidism were excluded from the study.

Ethics approval

Ethical clearance was obtained from the Institutional Ethics Committee with the approval number KIMSDU/IEC/07/2022.

Methodology

A total of 350 women were screened in their first and second trimesters. Written informed consent was taken from the patients on their first visit to the OPD. Their clinical details, including an extensive history, age, results of physical examinations and obstetric information, were meticulously documented. Samples for TSH and FT4 estimation were collected from the fasting patients in red vacutainer (plain bulb) and then analyzed using the TOSOH AIA 360 immunoassay analyzer (Tosoh India Pvt. Ltd., Mumbai, India). If serum TSH was abnormal, FT4 and free triiodothyronine (FT3) were estimated. The participants were grouped as patients with euthyroidism, overt hypothyroidism and subclinical hypothyroidism, according to their thyroid function results.

According to American Thyroid Association (ATA) guidelines 2017, the upper limit of normal cut-off for TSH levels in the first trimester should be obtained by deducting 0.5 mIU/L from the pre-pregnancy TSH value. If this value is unknown, then 4.0 mIU/L should be considered as the cut-off value [6]. As pre-pregnancy TSH levels were not available, the cut-off value for TSH was taken as 4.0 mIU/L in the present study. The TSH levels were reassessed at 16, 20, and 32 weeks, ensuring that any adjustments in treatment could be made promptly. The criteria for subclinical and overt hypothyroidism were TSH levels between 2.5-10 mIU/L and greater than 10 mIU/L, respectively. If TSH levels in patients with hypothyroidism were ≤3 mIU/L at 32 weeks of gestation, they were considered as adequately treated. On the other hand, if the levels were ≥3 mIU/L, they were considered as inadequately treated. All patients were monitored up to the point of delivery, allowing for comparison of outcomes across the three groups. Maternal outcomes of interest included preeclampsia, eclampsia, anemia, gestational hypertension, gestational diabetes mellitus (GDM), spontaneous miscarriage, preterm labor, oligohydramnios, etc. [7]. Similarly, fetal outcomes included intrauterine growth restriction (IUGR), LBW, and fetal distress [8].

Statistical analysis

The data was compiled in Microsoft Excel (Microsoft Corp., Redmond, WA, US) and analyzed using IBM SPSS Statistics for Windows, Version 19 (Released 2010; IBM Corp., Armonk, New York, US). The analysis included a comparison of outcomes between groups categorized as those with euthyroidism, overt hypothyroidism, and subclinical hypothyroidism. Variables were evaluated using Pearson chi-squared test or ANOVA test, as appropriate. Pearson chi-squared test was used to analyze the relationship between two categorical variables, for example, mode of delivery in women with hypothyroidism and euthyroidism. ANOVA test was used to compare the means of more than two groups, for example, while comparing the TSH levels at 16, 20, and 32 weeks of gestation. The p value was calculated for each parameter and p<0.05 was considered as statistically significant.

## Results

Out of the 350 patients (100%), 311 (88.86%) were classified with euthyroidism and 39 (11.14%) with hypothyroidism. Twenty-eight patients (8%) had subclinical hypothyroidism whereas 11 (3.14%) had overt hypothyroidism (Table 1). 

**Table 1:** Distribution of cases according to prevalence n=number of cases

Cases	Frequency (%)
Euthyroidism	311 (88.86%)
Subclinical hypothyroidism	28 (8.0%)
Overt hypothyroidism	11 (3.14%)
Total (n)	350 (100%)

Out of the 39 cases with hypothyroidism, 17 (43.59%) were between 26 and 30 years old while 12 (30.77%) were between the ages of 18 and 25 years. The youngest patient was 19 years old and the oldest was 40. The majority of patients with hypothyroidism i.e. 35 patients (89.75%) weighed more than 50 kgs. The maximum weight of the patients diagnosed with overt hypothyroidism was 98 kgs.

Twenty-one (53.85%) multigravida and 18 (46.15%) primigravida patients were diagnosed with hypothyroidism. There was a higher incidence of hypothyroidism in multigravida patients in the present study. The majority of patients with euthyroidism (63.03%) were primigravida (Table 2). Most of the patients underwent screening at less than 10 weeks of gestational age and a majority of them (64.10%) were diagnosed with hypothyroidism in that period.

**Table 2:** Distribution of cases based on their obstetric codes n=number of cases, primi=primigravida, multi=multigravida, LSCS=lower segment caesarean section.

Description	Women with hypothyroidism, n (%)	Women with euthyroidism, n (%)
Primi	18 (46.15%)	196 (63.03%)
Multi with previous normal delivery	06 (15.39%)	71 (22.83%)
Multi with previous LSCS	15 (38.46%)	44 (14.14%)
Total	39 (100%)	311 (100%)

Out of the 39 patients with hypothyroidism, 17 (43.58%) had TSH levels between 4.2 and 10 mIU/L, 28 (71.79%) had subclinical hypothyroidism (TSH 2.5-10 mIU/L), and 11 (28.21%) had overt hypothyroidism (TSH>10 mIU/L; Table 3). Patients with deranged TSH values (TSH>4 mIU/mL) underwent FT4 testing. Majority of the patients tested had normal T4 levels suggesting that subclinical hypothyroidism was more prevalent than overt hypothyroidism.

**Table 3:** Distribution of cases according to TSH Levels (mIU/L) n= number of cases, TSH= thyroid stimulating hormone

TSH level	Women with hypothyroidism, n (%)	Women with euthyroidism, n (%)
<2.5	00 (0%)	299 (96.15%)
2.5-4.2	11 (28.21%)	12 (3.85%)
4.2-10	17 (43.58%)	00 (0%)
>10	11 (28.21%)	00 (0%)
Total	39 (100%)	311 (100%)

The 39 patients diagnosed with hypothyroidism were prescribed levothyroxine and four (10.25%) of them achieved normal TSH levels by 20 weeks. However, 32 (82.05%) patients still had elevated TSH levels, requiring an increase in their dose. The dose of levothyroxine was decided by the treating clinician as deemed necessary according to the weight of the patient, trimester and the target TSH value. By 32 weeks of gestation, 13 (33.33%) patients had normalized their TSH levels, while the remaining needed further dosage adjustments.

Additionally, four (10.25%) patients experienced spontaneous miscarriage before reaching 32 weeks of gestation. Patients who started levothyroxine before 10 weeks of gestation and achieved normal TSH levels by 32 weeks were considered adequately treated. Those who did not reach normal TSH levels despite aggressive treatment were classified as inadequately treated. At the end of 32 weeks, out of the 39 patients with hypothyroidism, 23 (58.98%) patients were adequately treated and 16 (41.02%) were inadequately treated.

**Figure 1:**
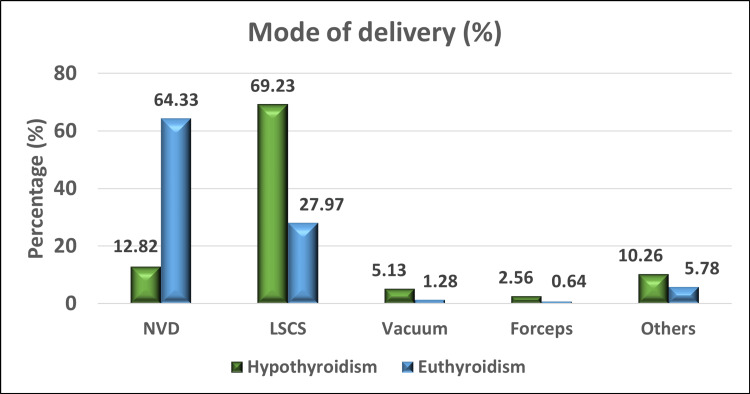
Graph showing mode of delivery among women with hypothyroidism and euthyroidism NVD=normal vaginal delivery, LSCS=lower segment caesarean section.

Twenty-seven (69.23%) out of the 39 patients with hypothyroidism underwent lower segment caesarean section (LSCS), while five (12.82%) had a normal vaginal delivery. In contrast, among the women with euthyroidism, majority of them i.e. 200 (64.33%) had normal vaginal deliveries, with 87 (27.97%) undergoing LSCS (Figure 1). The reasons for LSCS in the patients with hypothyroidism included preeclampsia, preterm labor, GDM, gestational hypertension, and fetal distress. This indicates a higher rate of LSCS in women with hypothyroidism compared to those with euthyroidism.

Complications were observed in all 16 (100%) patients in the inadequately-treated group with hypothyroidism, whereas only seven of the adequately-treated patients (30.4%) experienced complications. Majority of the adequately-treated patients i.e. 16 (69.6%) delivered without complications.

The present study showed a higher incidence of spontaneous abortions i.e. three (18.75%) in patients with overt hypothyroidism who were inadequately treated. One of the patients with overt hypothyroidism in the adequately-treated group spontaneously aborted and the fetus was diagnosed with Dandy Walker syndrome. The prevalence of preterm labor was higher in inadequately-treated patients than in the adequately-treated group [two (12.5%) vs. one (4.34%)]. Maternal complications such as preeclampsia, gestational hypertension, GDM and low hemoglobin showed increased prevalence in the inadequately-treated than in the adequately-treated patients. Fetal outcomes such as LBW, IUGR, and fetal distress was also more common in the former than in the latter (Table 4).

**Table 4:** Distribution of pregnancy outcomes in the adequately- and inadequately-treated group of patients with hypothyroidism n=number of cases, GDM=gestational diabetes mellitus, IUGR=intrauterine growth retardation, Hb=hemoglobin

Outcome	Frequency (n)	Adequately treated (%)	Frequency (n)	Inadequately treated (%)
Spontaneous miscarriage	1	4.34	3	18.75
GDM	0	00	1	6.25
Preeclampsia	1	4.34	2	12.5
Oligohydramnios	0	00	1	6.25
Preterm labour	1	4.34	2	12.5
IUGR	0	00	1	6.25
Low birth weight	2	8.7	2	12.5
No complications	16	69.6	0	00
Decreased Hb	1	4.34	2	12.5
Gestational hypertension	1	4.34	1	6.25
Fetal distress	0	00	1	6.25
Total	23	100	16	100

When the inadequately-treated patients with hypothyroidism were compared to those with euthyroidism, the latter group developed fewer complications than the former. Three (18.75%) patients in the former group had spontaneous miscarriages vs. only one (4.8%) in the latter. Similarly, 12.5% of the inadequately-treated patients developed preeclampsia vs. 8.68% of the patients with euthyroidism. Complications like GDM, preeclampsia, oligohydramnios, preterm delivery, IUGR, LBW babies, and fetal distress were more commonly seen in inadequately-treated patients with hypothyroidism compared to patients with euthyroidism. This indicated a significant association between inadequately-treated hypothyroidism and poor pregnancy outcomes (Table 5).

**Table 5:** Distribution of pregnancy outcomes in patients with euthyroidism and those with inadequately-treated hypothyroidism DM=diabetes mellitus, IUGR=intrauterine growth retardation, PROM=premature rupture of membrane, IUFD=intrauterine fetal demise, Hb=hemoglobin

Outcomes	Patients with euthyroidism	Patients with Inadequately-treated hypothyroidism
Spontaneous miscarriage	15 (4.8%)	03 (18.75%)
Gestational DM	16 (5.15%)	01 (6.25%)
Preeclampsia	27 (8.68%)	02 (12.5%)
Oligohydramnios	08 (2.57%)	01 (6.25%)
Preterm labour	18 (5.79%)	02 (12.5%)
IUGR	8 (2.57%)	01 (6.25%)
Low birth weight	13 (4.19%)	02 (12.5%)
No complications	139 (44.70%)	00
PROM	14 (4.50%)	00
IUFD	01 (0.32%)	00
Decreased Hb	34 (10.93%)	02 (12.5%)
Gestational hypertension	15 (4.83%)	01 (6.25%)
Ectopic pregnancy	01 (0.32%)	00
Missed abortion	02 (0.64%)	00
Fetal distress	00	01 (6.25%)

## Discussion

The purpose of this study was to follow the pregnancy outcomes in women with hypothyroidism and to check if complications developed after providing appropriate treatment. The total number of pregnant women included in this study was 350. All the women were screened at their first visit during the first or second trimester. Those who had an elevated TSH level were further tested for FT4 levels. As per the ATA guidelines, the cut-off level of 4.0 mIU/L for TSH in the first trimester was considered [6]. Depending on their thyroid function, patients were diagnosed and categorized into three groups: euthyroidism, overt hypothyroidism, and subclinical hypothyroidism [8]. 

All the women who were diagnosed with hypothyroidism were started on levothyroxine treatment as deemed necessary. TSH levels were evaluated at 16, 20, and 32 weeks of gestation, ensuring that any adjustments in treatment could be made promptly. According to the gestational age at the time of screening and the initiation of treatment, i.e. less or more than 10 weeks, and the TSH levels at the end of 32 weeks, the patients were classified as adequately (TSH<3 mIU/L) or inadequately treated (TSH>3 mIU/L). Both groups were followed up till delivery and the development of maternal and fetal complications was noted.

Out of the 350 cases, 39 (11.14%) had hypothyroidism. Among these, 28 (8.0%) were diagnosed with subclinical hypothyroidism and 11 (3.14%) with overt hypothyroidism. The prevalence of hypothyroidism (11.14%) in this study was based on the TSH cut-off value of 4.0 mIU/L in the first trimester. This was similar to the studies conducted by Mahadik et al. (2020) [9], Yadav et al. (2021) [10], Kukreja et al. (2022) [11], Mishra et al. (2024) [12] and Sharma et al. (2024) [13].

Age distribution

In our study, the incidence of hypothyroidism was more common in women between 26 and 30 years of age (mean 26.9 years). The youngest patient with hypothyroidism was 19 years old and the oldest was 40 years old. This suggests that there is an increased prevalence of hypothyroidism as the age increases [8]. Similar findings were observed in the studies conducted by Singh et al. (2018) [14] and Kumar et al. (2023) [15]. 

Body weight distribution

In this study, a majority of patients with hypothyroidism i.e. 35 (89.75%) weighed more than 50 kgs. The maximum weight of the patients diagnosed with overt hypothyroidism was 98 kgs. This indicated that the incidence of maternal hypothyroidism increases with an increase in weight of the patient. Our finding was in concordance with Knøsgaard et al. (2022) [16] and Mainali et al. (2023) [17]. 

Obstetric code

There was a higher incidence of hypothyroidism in multigravida patients in our study, which was concordant with the finding of studies by Singh et al. (2018) [14], Jain et al. (2021) [18] and Priyadarshini et al. (2024) [19].

Gestational age at diagnosis

The majority of patients i.e. 25 (64.10%) were diagnosed with hypothyroidism at less than 10 weeks of gestational age which was in concordance with the studies by Jain et al. (2021) [18] and Mainali et al. (2023) [17]. Upon screening, out of the 350 patients studied, 39 (11.14%) cases of hypothyroidism and 311 (88.85%) of euthyroidism were diagnosed. Twenty-eight of the 39 patients (71.79%) had subclinical hypothyroidism with TSH levels between 2.5-10 mIU/L and 11 (28.21%) had overt hypothyroidism with TSH levels >10 mIU/L. Thus, subclinical hypothyroidism was more commonly observed than overt hypothyroidism in this study. Similar findings were noted in the studies by Kukreja et al. (2022) [11], Kumar et al. (2023) [15], and Sharma et al. (2024) [13].

T4 levels

In the present study, all patients with abnormal TSH values were tested for FT4. It was observed that most patients had normal T4 levels, suggesting that subclinical hypothyroidism was more common than overt hypothyroidism. Overt hypothyroidism is rare as it associated with anovulation and infertility [20]. Similar findings were observed in the studies by Mahadik et al. (2020) [9], Yadav et al. (2021) [10], and Vamja et al. (2024) [21].

Repeat TSH levels at different weeks

Majority of patients who were initiated on levothyroxine therapy responded well, with lowering of TSH levels over a period of time. Rao et al. (2019) observed that levothyroxine therapy significantly decreased fetal and maternal complications [22]. Compared to patients with euthyroidism, a larger proportion of patients with hypothyroidism underwent LSCS [27 out of 39 (69.23%)]. Additionally, five patients (12.82%) had normal vaginal deliveries, three patients (7.69%) had assisted deliveries using vacuum or forceps, and four patients (10.26%) experienced spontaneous miscarriage. Indications for LSCS in patients with hypothyroidism were preeclampsia, preterm labor, GDM, gestational hypertension, and fetal distress. In the studies conducted by Kumar et al. (2023) [15] and Priyadarshini et al. (2024) [19], 62.1% and 52% of patients with hypothyroidism underwent LSCS, respectively. Similar results were found in our study, where LSCS was the more frequently observed mode of delivery for patients with hypothyroidism. This finding aligns with the research conducted by Mahadik et al. (2020) [9] and Raju et al. (2020) [23].

Treatment adequacy and complications

If patients who were diagnosed and started on levothyroxine before 10 weeks of gestation had normal TSH levels at the end of 32 weeks, then they were considered adequately treated. On the other hand, those who failed to reach normal TSH levels despite aggressive treatment were classified as inadequately treated.

Out of the 39 patients with hypothyroidism in our study, 23 (58.98%) were adequately treated and 16 (41.02%) were inadequately treated. Out of the former group, seven patients developed complications whereas all from the latter developed complications.

Uncorrected hypothyroidism in pregnancy has adverse effects on fetal and maternal outcomes [24]. Therefore, early and adequate treatment leads to successful pregnancy outcomes [25,26]. Similar findings were observed by Jain et al. (2021) [18], Priyadarshini et al. (2024) [19], and Vamja et al (2024) [21].

Comparison of outcomes between adequately- and inadequately-treated patients with hypothyroidism

The present study showed a higher incidence of spontaneous abortions in patients with overt hypothyroidism who were inadequately treated (18.75%) compared to those who were adequately treated (4.34%). Similar findings were observed by Nayak et al. (2019) where 17.9% of inadequately-treated patients underwent abortions [27]. In our study, one of the patients with overt hypothyroidism in the adequately-treated group had a spontaneous abortion and the fetus was diagnosed with Dandy Walker syndrome. The prevalence of preterm labor was three times higher in the inadequately-treated patients than in the adequately-treated patients (12.5 vs. 4.34%). Similar findings were noted in the studies by Knøsgaard et al. (2022) [16] and Suman et al. (2024) [28], where preterm labor was observed in 11% and 12.14% of cases, respectively. Maternal complications such as preeclampsia, gestational hypertension, GDM and low hemoglobin had increased prevalence in the inadequately-treated group compared to the adequately-treated one, with majority of the patients in the latter having no complications. This finding was concordant with studies by Kumar et al. (2023) [15], Mishra et al. (2024) [12], and Sharma et al. (2024) [13]. Fetal outcomes such as LBW, IUGR, and fetal distress were also more common in inadequately-treated patients than in adequately-treated ones. This was also observed in the studies by Mahadik et al. (2020) [9], Kumar et al. (2023) [15], and Vamja et al. (2024) [21].

Comparison of outcomes between inadequately-treated patients with hypothyroidism and those with euthyroidism

In the present study, 18.75% of the inadequately-treated patients with hypothyroidism had a spontaneous miscarriage whereas it occurred in only 4.8% of patients with euthyroidism. Similar findings were noted in the studies by Monika et al. (2022) [8] and Knøsgaard et al (2022) [16]. A greater proportion of the inadequately-treated patients developed preeclampsia (12.5%) compared to those with euthyroidism (8.68%). These were concordant with the findings of Vamja et al. (2024) [21] where preeclampsia was seen in 18% of patients with hypothyroidism and in 6% of patients with euthyroidism. Similarly, 6.25% of the inadequately-treated patients developed oligohydramnios compared to 2.57% of the patients with euthyroidism. Thus, it was more common in the former group than in the latter. The study by Monika et al. (2022) [8] showed similar findings. Around 12.5% of the inadequately-treated patients with hypothyroidism had preterm labor while only 5.79% of patients with euthyroidism had a preterm delivery. Chaudhary et al. (2021) [29] also showed a similar finding with preterm delivery occurring in 13.3% of indequately-treated cases. Around 6.25% of the inadequately-treated patients in our study had IUGR whereas it was observed in only 2.57% of patients with euthyroidism, a finding similar to that seen in Monika et al (2022) [8], where it occurred in 5.7% of inadequately-treated cases.

In this study, 12.5% of the inadequately-treated patients with hypothyroidism gave birth to LBW babies compared to just 4.19% in the group with euthyroidism. This finding is similar to rate of LBW babies (12.7%) reported by Kumar et al. (2023 [15]. Fetal distress was more common in the inadequately-treated patients with hypothyroidism than in those with euthyroidism. This was concordant with the study by Mishra et al. (2024) [12]. No case of intrauterine fetal demise was observed in the inadequately-treated patients in this study, which was concordant with the study by Mahadik et al. (2020) [9]. Thus, a majority of the inadequately-treated patients developed more complications like GDM, pre-eclampsia, IUGR, oligohydramnios, preterm deliveries, and LBW babies as compared to those with euthyroidism.

## Conclusions

Pregnancy is a state of increased metabolism that can mask symptoms of hypothyroidism. In the current study, hypothyroidism was observed in 11.14% (n=39) of cases, with subclinical hypothyroidism being more prevalent than overt hypothyroidism. LSCS was the most common mode of delivery among women with hypothyroidism. Adverse maternal and fetal outcomes like preeclampsia, preterm labor, anemia, GDM, spontaneous abortion, oligohydramnios, IUGR, and LBW babies were noted in the inadequately-treated patients with hypothyroidism. Hence, early diagnosis and adequate treatment of maternal hypothyroidism leads to successful pregnancy outcomes. Therefore, universal thyroid screening during pregnancy is recommended to prevent maternal and fetal complications.
